# AI-Blue-Carba: A Rapid and Improved Carbapenemase Producer Detection Assay Using Blue-Carba With Deep Learning

**DOI:** 10.3389/fmicb.2020.585417

**Published:** 2020-11-20

**Authors:** Ling Jia, Lu Han, He-Xin Cai, Ze-Hua Cui, Run-Shi Yang, Rong-Min Zhang, Shuan-Cheng Bai, Xu-Wei Liu, Ran Wei, Liang Chen, Xiao-Ping Liao, Ya-Hong Liu, Xi-Ming Li, Jian Sun

**Affiliations:** ^1^National Risk Assessment Laboratory for Antimicrobial Resistance of Animal Original Bacteria, South China Agricultural University, Guangzhou, China; ^2^Guangdong Provincial Key Laboratory of Veterinary Pharmaceutics Development and Safety Evaluation, South China Agricultural University, Guangzhou, China; ^3^College of Mathematics and Informatics, South China Agricultural University, Guangzhou, China; ^4^Public Health Research Institute Tuberculosis Center, New Jersey Medical School, Rutgers University, Newark, NJ, United States; ^5^Jiangsu Co-Innovation Center for Prevention and Control of Important Animal Infectious Diseases and Zoonoses, Yangzhou, China

**Keywords:** carbapenemase-producing gram-negative bacteria, rapid detection, Blue-Carba, deep learning, OD value

## Abstract

A rapid and accurate detection of carbapenemase-producing Gram-negative bacteria (CPGNB) has an immediate demand in the clinic. Here, we developed and validated a method for rapid detection of CPGNB using Blue-Carba combined with deep learning (designated as AI-Blue-Carba). The optimum bacterial suspension concentration and detection wavelength were determined using a Multimode Plate Reader and integrated with deep learning modeling. We examined 160 carbapenemase-producing and non-carbapenemase-producing bacteria using the Blue-Carba test and a series of time and optical density values were obtained to build and validate the machine models. Subsequently, a simplified model was re-evaluated by descending the dataset from 13 time points to 2 time points. The best suitable bacterial concentration was determined to be 1.5 optical density (OD) and the optimum detection wavelength for AI-Blue-Carba was set as 615 nm. Among the 2 models (LRM and LSTM), the LSTM model generated the higher ROC-AUC value. Moreover, the simplified LSTM model trained by short time points (0–15 min) did not impair the accuracy of LSTM model. Compared with the traditional Blue-Carba, the AI-Blue-Carba method has a sensitivity of 95.3% and a specificity of 95.7% at 15 min, which is a rapid and accurate method to detect CPGNB.

## Introduction

Antimicrobial resistance (AMR) poses a serious global threat to human, animal, and environment health, as multidrug resistant bacteria continue to emerge and spread worldwide. Carbapenems are one of the last-resort antibiotics to treat infections caused by multidrug-resistant Gram-negative pathogens. Carbapenem resistance in Gram-negative bacteria is primarily due to the production of various carbapenemases, which leaves the clinicians with limited therapeutic options. Carbapenemase-producers showed broad spectrum enzyme activity for various β-lactam substrates, and were associated with resistance to other antibiotic classes, and demonstrated rapid transmission in healthcare facilities, animals and the environments (Codjoe and Donkor, 2017). Notably, carbapenemase genes are frequently located on mobile genetic elements and plasmids, therefore facilitating the horizontal of resistance to other bacteria (Dortet et al., 2014; Nordmann and Poirel, 2014). It is of paramount importance to develop reliable methods for rapid detection and characterization of carbapenemase-producers.

Different phenotypic and molecular-based methods have been used to identify these carbapenemase producers. For known mechanisms, molecular methods of gene detection are usually fast and accurate. However, molecular detection of carbapenemase genes can be costly and may require substantial expertise, and more importantly they fail to detect unknown or novel carbapenemase genes (Stuart et al., 2012). A solution to this problem is the detection of carbapenem enzymatic degradation, using Matrix Assisted Laser Desorption Ionization-Time of Flight Mass Spectrometry (MALDI-TOF MS) (Yu et al., 2018); or by chromogenic agar or UV spectrophotometry (e.g., Carba NP and Blue Carba) (Bernabeu et al., 2012); or the rapid Carbapenem Inactivation Method (Muntean et al., 2018).

In 2012, Nordmann et al. developed the Carba NP test which is based on visual monitoring of medium acidification of a mixture containing bacterial cells, a carbapenem and the pH indicator phenol red (Dortet et al., 2012). However, some subtle color variations could be hard to differentiate by visual interpretation. This method was then adapted to microtiter plates and spectrophotometric measurement of optical density and interpreted using a pre-programmed Excel spreadsheet (Surre et al., 2018). However, the assay was not comprehensive enough and the data analysis of this assay is still not straightforward.

In this study, a deep learning approach was used to predict carbapenemase production, taking into consideration the similarity in the OD value distributions at different time points, instead of only the best hit. Deep learning has been proven to be the most powerful machine learning approach to date for many applications, including image processing (LeCun et al., 2015), biomedical signaling (Tabar and Halici, 2017), speech recognition (Hinton et al., 2012), and genomic related analysis, such as the predicting antibiotic resistance genes from metagenomic data (Pan and Shen, 2017; Li et al., 2018). Particularly in the case of predicting new genetic markers, the deep learning model surpasses all known binding site for prediction approaches (Drouin et al., 2016).

To the best our knowledge, this is the first time that contact the carbapenemase detection method with Deep learning. Here we describe the AI-Blue-Carba test, an improved variant of Blue-Carba to determine carbapenemase producers using a uniform standard.

## Materials and Methods

### Sample Collection and Bacterial Strains

In this study, we mainly collected fecal samples from animals (anal swabs and feces). These fecal samples were randomly collected from pigs, chickens, ducks, and goose, if possible, the soil, dust, sewage and vegetable samples were also collected. These samples were screened from 14 animal farms (pig farms, *n* = 5; chicken houses, *n* = 5; duck farms, *n* = 3; goose farms, *n* = 1) in 6 provinces (Guangxi, Guangdong, Heilongjiang, Jiangsu, Jiangxi, and Zhejiang provinces) in China. In total, 498 strains were collected from June 2016 to Nov 2017 and subjected to selection onto MacConkey (MAC) agars containing meropenem (1 mg l-1). In order to enrich the diversity of carbapenem-resistant strains and genes, we also collected some strains from human and flower sources. Sixty clinical isolates were collected from the hospitals of Guangdong and Shandong provinces. In addition, 273 strains were isolated from flowers including carnations, roses and lilies which were collected from Mar 2018 to Apr 2018 in Guangzhou Flower Market and selected on MAC plates without any antibiotics.

We utilized 130 among the 831 collected strains from different sources above, including 49 isolates from animal anal swabs and feces samples which were characterized, 60 clinical isolates collected from two hospitals in Guangdong and Shandong provinces, and then 21 isolates from flowers. Carbapenemase genes were characterized by PCR and Sanger sequencing (Rahman et al., 2013). Strains from different Enterobacteriaceae species (*E. coli, Klebsiella pneumoniae*, *Citrobacter freundii, Enterobacter cloacae*, etc.) as well as *Pseudomonas spp.* were included. We identified 107 carbapenem-resistant strains able to produce at least one carbapenem-hydrolyzing β-lactamase, whereas the remaining 23 carbapenem-susceptible strains were carbapenemase negative (Table 1). The MICs for ertapenem, meropenem, and imipenem were determined by agar dilution and interpreted according to the Clinical and Laboratory Standards Institute guidelines (CLSI, 2018).

**TABLE 1:** Results of carbapenemase and non-carbapenemase producers’ PCR and MIC.

**Species**	**MIC(μg/ml)**			**No. of isolates with a positive test/no. of isolates tested for 2 h**		
		
	**Carbapenemase content**	**MEM**	**IPM**	**ERT**	**AI-Blue-Carba**	**Blue-Carba**
**Carbapenemase producers*E. coli* (47)**	NDM-5(43)	1–64	1- >64	>64	41/43	41/43
	NDM-1(4)	4–8	8–16	16–64	4/4	4/4
***Pseudomonas putida*(7)**	VIM-2(6)	≥64	8-≥64	>64	6/6	6/6
	IMP-4(1)	>64	8	>64	1/1	1/1
***Klebsiella pneumoniae* (44)**	NDM-1(15)	2–32	2–32	8- >64	13/15	13/15
	NDM-5(4)	2–64	4- >64	16–64	4/4	4/4
	NDM-1 + IMP-4(2)	32	8- ≥64	>64	1/1	1/1
	KPC-2(23)	32	>64	>64	23/23	23/23
***Citrobacter freundii* (1)**	NDM-1(1)	16	8	64	1/1	1/1
***Enterobacter cloacae* (5)**	IMP-4(1)	8	16	64	1/1	1/1
	NDM-1(3)	16	16–32	32- >64	3/3	3/3
	VIM-1(1)	1	2	2	1/1	1/1
***Providencia rettgeri* (4)**	NDM-1(4)	2- ≥64	4	8	4/4	4/4
***Enterobacter mucus* (2)**	NDM-1(2)	32	32	>64	2/2	2/2
***Pseudomonas aeruginosa* (1)**	NDM-5(1)	64	>64	>64	1/1	1/1
**Non-carbapenemase producers**						
***E. coli (10)***	CTX-M	<0.0625	<0.0625–4	<0.0625–2	0/10	0/10
***Citrobacter freundii (8)***	CTX-M	<0.0625	<0.0625–4	<0.0625–2	0/8	0/8
***Enterobacter cloacae (4)***	CTX-M	<0.0625	<0.0625–4	<0.0625–2	0/4	0/4
***Providencia rettgeri (1)***	CTX-M	<0.0625	<0.0625–4	<0.0625–2	0/1	0/1

*MEM, meropenem; IPM, imipenem; ERT, ertapenem.*

### Blue-Carba Test

The Blue-Carba test was performed and interpreted as previously described (Pires et al., 2013). Briefly, 5 μL loopfuls of bacteria cultured on Mueller-Hinton agar (HuanKai, Guangzhou, China) were suspended in 0.04% bromothymol blue (Macklin, Shanghai, China) solution containing (test) or lacking (control) 3 mg/mL imipenem (MedChemExpress, New Jersey, United States) and 0.1 mM ZnSO4 (Damao, Tianjin, China). Color changes were registered after incubation at 37°C for 2 h. The result was considered positive when the solution containing imipenem became green or yellow and differed from the negative control. The result was negative if the solution lacking antibiotic presented the same or a stronger color change as the solution containing imipenem. A previously characterized NDM-5 producer (CQ02-121) was used as positive control (Sun et al., 2016) and a test tube containing only bacterial inoculum (*E. coli* ATCC 25922) and Blue-Carba solution was used as negative control for each isolate tested.

### Experiment Condition of AI-Blue-Carba

In order to overcome the limitation of lower the sensitivity with visual interpretation, we used the OD values to indicate the color’s change of the Blue-Carba result. To obtain a stable OD value, the optimum wavelength for detection of CPGNB was determined using a wavelength scan of test solutions generated using known carbapenemase producers up to 2 h in a Multimode Plate Reader (PerkinElmer, Hamburg, Germany) to obtain the absorbance maxima corresponding to yellow and blue (negative control).

Bacteria were diluted in 500 μL phosphate buffered saline (PBS, pH 7.4) and distilled water, respectively, the OD600 nm was adjusted to 1.0, 1.5, and 2.0 and the 100 μL of the bacteria suspension was used for testing. The wells were scanned and the OD was recorded every 5 min for 2 h at 37°C in the plate reader.

### Deep Learning

The problem of distinguishing carbapenemase and non-carbapenemase producers based on the OD values and the results of the Blue-Carba test can be formalized as a machine learning or supervised learning question. It is assumed that a data sample *S* that contains *m* machine learning examples were given at the beginning. These examples are pairs (*x*,y), where *x* represents OD values detected over time and y is a label that corresponds to one of the two possible results (positive or negative). In addition, we assume that *x* ∈ {*a*_1_,*a*_2_…*a*_13_}which corresponds to the set of all 13 OD values and thaty ∈ {0,1}. Label y = 1 is assigned to the carbapenemase producers group and y = 0 is assigned to the non-carbapenemase producers group. We assumed the examples in *S* are drawn independently from an unknown and fixed data generating distribution *D*, resulting in the equation of S ^def^ = {(*x*_1_,*y*_1_),…(*x*_*m*_,*y*_*m*_)}∼*D^m^*.

Usually, learning algorithms are designed to learn from a vector representation of the data. In order to learn from detected OD values, a function∅:{*x*_1_,*x*_2_,…*x*_*m*_}*→*R^d^* is defined, which takes time as input and maps it to some *d* dimensional vector space. Then a learning algorithm can be applied to the set S′ ^def^ = {∅(*x*_1_),*y*_1_,…∅(*x*_*m*_,*y*_*m*_)} to obtain a model h:*R^d^*→{0,1}. The model is a function which maps the feature representation of OD values over time to the associated results of the carbapenemase producers. Our objective is to achieve a model *h* that has a good generalization performance, i.e., that minimizes the probability, R(*h*), of making a prediction error for any example drawn according to the distribution *D*, where

(1) R(h)def=Pr[h(∅(x))≠y](x,y)∼DR

### Construct Models

The LSTM (Long Short-Time Memory) cells store and access information over long periods of time using multiplicative gates (Hochreiter and Schmidhuber, 1997). It retains useful long-term information through the threshold mechanism and removes useless short-term information to realize the mining of timing information. In this work, the rules were individual units that detect the carbapenemase-producer using OD values over time. These rules are Boolean-valued, i.e., the output is either positive or negative. The models learned by the LSTM are logical combinations of such rules, which can be conjunctions (logical-AND) or disjunctions (logical-OR). To predict the results of detection of carbapenemase producers using OD values over time, each rule in the model was evaluated and the results were aggregated to obtain the prediction.

Real-time prediction of data traffic requires continuous data input and learning. The dataset was therefore split into a training set (80% of the OD values) to construct the LSTM model that can output a *y* value randomly (0 or 1 which represent the positive or negative results of carbapenemase producer) if input a group of OD values. In addition, a separate testing set (20% of the data) were used with the results of Blue-Carba to validate and evaluate LSTM’s prediction accuracy. In this study, we used a 10-fold cross validation to test the prediction model and select the best hyperparameter (Webb et al., 2019).

In order to display data characteristics to more accurately predict the carbapenemase-producing strains, we compared the LSTM with LRM (Linear Regression model). The LRM is a linear approach to find the relationship between a scalar response and one or more explanatory variables (Kumaresan and Riyazuddin, 2007). In this paper, LRM modeled the relation between OD values and Blue-Carba test.

### Evaluation Metrics

We evaluated the performance of the two models using the confusion matrix assessment method. The prediction quality was evaluated by the following evaluation metrics: The ROC curve describes the classifier’s True Positive Rate (TPR, the ratio of the number of positive samples correctly classified by the classifier to the total number of positive samples) and False Positive Rate (FPR, the number of negative samples that the classifier misclassified accounts for the total negative samples) Ratio of the number). Recall [Equation 2] shows the number of correct positive results divided by the number of all relevant samples that were identified as positive. Precision [Equation 3] expresses the number of correct positive results divided by the number of positive results predicted by the classifier. F1 Score [Equation 4] indicates the Harmonic Mean between Precision and Recall that tells you how precise your models is. The possible outcomes of a classification model include: true positive, TP; false positive, FP; true negative, TN; false negative, FN.

(2) $$\text{Recall}=\frac{\text{TP}}{\text{TP}+\text{FN}}$$

(3) $$\text{Precision}=\frac{\text{TP}}{\text{TP}+\text{FP}}$$

(4) $$\text{F}\,1-\text{score}=\frac{2\,\text{TP}}{2\,\text{TP}+\text{FP}+\text{FN}}$$

### Model Simplification

**FIGURE 1:**
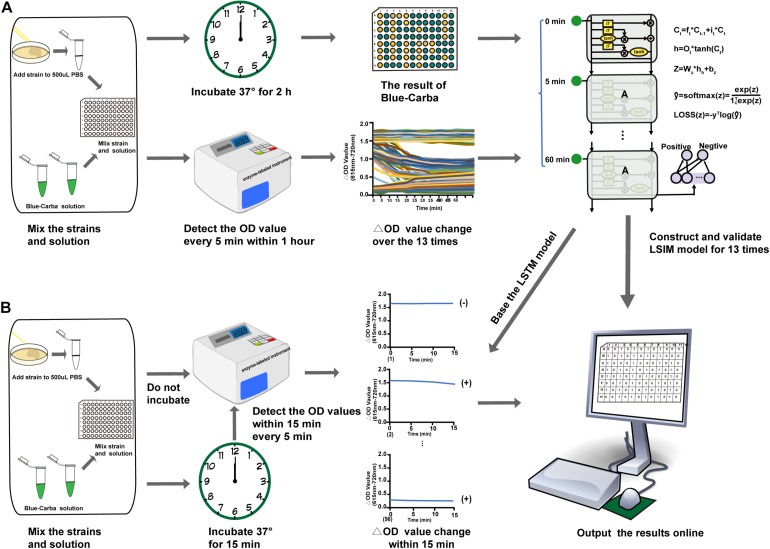
Rapid detection of carbapenemase producers by AI-Blue-Carba. **(A)**. Process to construct and validate the AI model; **(B)** Process to optimize the AI-Blue-Carba.

To achieve the purpose for rapid detection of a carbapenemase producer, we choose the OD values at 12 time points groups: 2 time points,3 time points, and so on 13 time points (Figure 1B) to optimize the best model from the above LSTM. We then evaluated the performance of the 12 models on the basis of the 12 sets of time points (OD value data set) using the above evaluation metrics to get the optimal detection time.

## Results

### Optimum Conditions for AI-Blue-Carba Test

The scanning results of positive and negative carbapenemase producers indicated a maximum absorption peak at 615 nm and this was chosen as the detection wavelength in following experiments (Supplementary Figure S1A). At the same time, we chose 720 nm as the detection wavelength of negative carbapenemase producers due to its stable absorption after 700 nm (Supplementary Figure S1B) to stabilize the OD values. Finally, the experimental data was the difference in OD (ΔOD) values between 615 and 720 nm.

We next examined differing bacterial concentrations and diluents at these wavelengths and found that the ΔOD values varied. The general trend in ΔOD values over time showed a more rapid decrease using the PBS diluent vs. ultrapure water at different bacterial concentrations (Supplementary Figures S2A–F). The weak carbapenemase producers showed a more rapid decrease in OD values as the bacterial concentration increased. However, when the bacterial concentration was set at 2.0 OD (at 600 nm), the strong carbapenemase producers generated with an ascending pattern over time (Supplementary Figures S2C,F). As such, we choose 1.5 OD (at 600 nm) for the bacterial concentration in PBS as the most appropriate testing condition for the AI-Blue-Carba test, due to more reliable stable OD values were acquired for both strong and weak carbapenemase producers.

We measured the OD values every 5 min up to 1 h (13 time points) using our 160 isolates (Table 1) to obtain the data characteristics. In brief, the trend in the ΔOD values of non-carbapenemase producers over time demonstrated a smooth linear pattern and ranged from 1.5 OD to 2.0 OD. The strong carbapenemase-producers generated a trend of ΔOD values over time that were also smooth lines, but the range was 0.2 OD – 0.4 OD while for weak carbapenemase producers, the trend of ΔOD values decreased gradually over time (Figure 1A).

### Construction and Evaluation of Deep Learning Model

**FIGURE 2:**
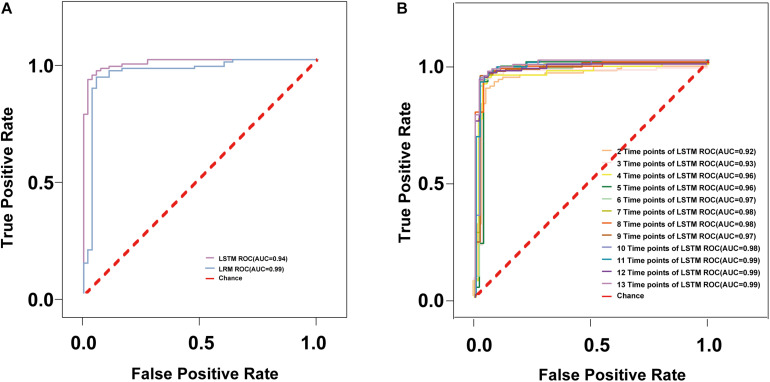
The ROC curves of different models. **(A)** The ROC curves are shown for the models trained with the multispecies data sets of Linear Regression model and LSTM model; **(B)** The ROC curves of different time points of LSTM.

In the following, we discuss the results of a 10-fold cross-validation study on the entire data set of 2 models. Across all cross-validation folds, predictor performance generalizes well to independent data. The degree of certainty of a read prediction can be measured by the prediction probability (see Methods). As Table 2 shows, the REC of LSTM is bigger (0.98) than LRM (0.62), other parameter values (ACC, REC, F1) of LSTM still higher than LRM. The model receiver operating characteristic (ROC) curves with the performances of LRM and LSTM (Figure 2A). The ROC-AUC of LSTM (0.99) was also better than LRM (0.94). Considering these factors, the LSTM model is the optimal model to detect the carbarpenemase producers.

**TABLE 2:** Prediction results for the majority prediction rule of four models.

**Model**	**ACC**	**F1**	**REC**	**PRE**
LRM	0.93	0.93	0.62	0.94
LSTM	0.98	0.98	0.98	0.98

*ACC, Accuracy; PRE, Precision; REC, Recall; F1, F1-score.*

### The Simplified Model

In order to determine the earliest time required to accurately detect carbapenemase producers, we used OD values with lesser time points and constructed and evaluated the models in a manner similar to that described above. To find a suitable model for specific application, we get the corresponding ACC, PRE, REC, and F1 values, sensitivity, specificity to adjust the threshold of the model, the ACC, PRE, REC, and F1 values of 12 optimized LSTM models were all >96% after 20 min in Table 3. In addition, the ROC-AUC values of the 12 time points group was >96% after 15 min (Figure 2B). Additionally, the 95.3% specificity and 95.7% sensitivity of AI-Blue-Carba were higher than Blue-Carba at 0–15 min. Consequently, we used the 0–15 min internal for rapid detecting the carbapenemase producers.

**TABLE 3:** Prediction results for the different time groups by LSTM model.

**Time Group**	**ACC**	**F1**	**REC**	**PRE**
0–5 min	0.87	0.87	0.88	0.89
0–10 min	0.90	0.92	0.92	0.92
0–15 min	0.93	0.93	0.94	0.94
0–20 min	0.96	0.96	0.96	0.96
0–25 min	0.97	0.97	0.97	0.97
0–30 min	0.98	0.98	0.98	0.98
0–35 min	0.97	0.97	0.97	0.97
0–40 min	0.97	0.97	0.97	0.97
0–45 min	0.97	0.97	0.97	0.97
0–50 min	0.97	0.97	0.96	0.97
0–55 min	0.97	0.97	0.97	0.97
0–60 min	0.98	0.98	0.98	0.98

**FIGURE 3:**
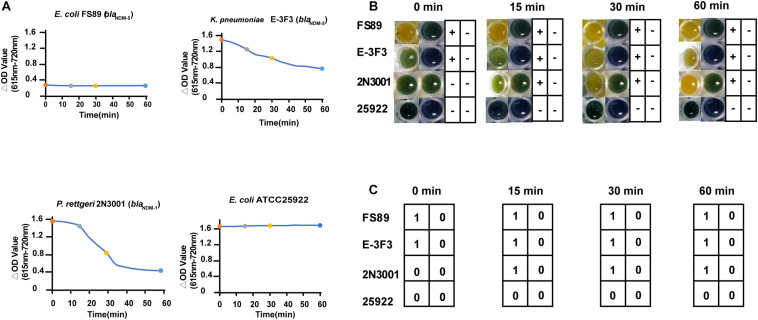
Comparison of the result of AI-Blue-Carba and Blue-Carba. **(A)** shows the ΔOD values’ change of different time points (within in 0 min, 0–15 min, 0–30 min, 0–60 min); **(B)** shows the results of carbapenamase producer by Blue-Carba; **(C)** shows the results of carbapenamase producer by AI -Blue-Carba within.

We examined some weak (*Klebsiella pneumonia* E-3F3 and *Citrobacter freundii* 2N3001), strong (*E. coli* FS89) and non-carbapenemase producers (*E. coli* ATCC 25922) using the Blue-Carba test. Isolates FS89 and ATCC 25922 could be detected in 0 min, while E-3F3 and 2N3001 could be accurately judged after 30 min but the confirmation of the final results took 2 h by Blue-Carba. The ΔOD values for FS89 and 25922 from 0–15 min, 0–30 min and 0–60 min were consistent with the trend of the 13-time points taken over 1 h. This indicated that the strong carbapenemase and the non-carbapenemase producers can be rapidly detected within 15 min. For E-3F3 and 2N3001, the ΔOD values for the 13-time points was relatively slow for the 0–15 min, 0–30 min, and 0–60 min intervals (Figure 3A). The colors change of Blue-Carba (Figure 3B) and the results of AI-Blue-Carba (Figure 3C) were consistent with the PCR results.

## Discussion

The rapid increase of carbapenem resistance in Gram-negative bacilli is of great concern worldwide (Zhang et al., 2018). Public health surveillance for a disease is traditionally viewed as the first step in disease prevention and data obtained from surveillance help to enforce public health action. Therefore, rapid and user-friendly assays are crucial.

To rapidly and efficiently detect carbapenemase producers, the Carba NP test was modified according to CLSI guidelines by measuring the *in vitro* hydrolysis of imipenem to produce a color change within 2 h (Dortet et al., 2012). The RAPID ECCARBA NP test could be useful for screening carbapenemase producers from colonized patients (Poirel and Nordmann, 2015). The commercially available β CARBA test is based on the change of color of an undisclosed chromogenic substrate in the presence of carbapenem-hydrolyzing activity. The test is simple to perform and interpret by non-specialized staff members (Mancini et al., 2017). In addition, the CarbAcineto NP test, which is rapid and reproducible, detects all types of carbapenemases including *Acinetobacter spp.* with a sensitivity of 94.7% and a specificity of 100%. Its use will facilitate its recognition and prevent its spread.

All these modified Carba NP tests have an obvious shortcoming that the color intensity is somewhat low, so we choose the LSTM model to correct this deficiency. Carbapenemase detection by spectrophotometric assays is a more accurate approach for the detection of carbapenemases and has excellent sensitivity and specificity for the Enterobacteriaceae. The results are usually comparable between different labs and were suggested to be implemented in national reference laboratories (Nordmann et al., 2012). Moreover, all the above methods can use these tools (OD values and the LSTM model) to improve their sensitivity and specificity.

A pioneering study illustrated the huge potential of using Big Data for epidemiology in which the epidemics can be detected early by tracking online queries on disease symptoms using social media such as Google Search and Twitter (Deiner et al., 2016). We introduce a “deep learning” approach to improve the objectivity and efficiency of detecting carbapenemase producers. The deep learning model can be applied to new data to make decisions after training, and decision making can involve detection, discrimination, and classification. Therefore, we trained the LRM and LSTM models using OD values generated by the Blue Carba test and compared the evaluation metrics of the four models. The results presented below could not be notably improved by further parameter tuning or feature selection efforts. An auxiliary assessment further shows that our classifiers are very robust. We chose LSTM to construct the website because it was optimized by the evaluation metrics independent of the values of the F1-scores. As we expected, its advantages are most pronounced for problems requiring the use of long-range contextual information. Consequently, LSTM has also been applied to various real-world problems, such as protein secondary structure prediction, music generation, reinforcement learning, speech recognition, and handwriting recognition. Furthermore, similar to any software systems, updating this classification system or model can be conducted at regular intervals whenever new dataset/information/evidence is available.

To achieve the aim of the rapid detection of the carbapenemase producers, we simplified the LSTM model by reducing detection times and chose 15 min as test interval. The deep learning analysis platform illustrates that ΔOD values change over time intuitively and then judges the strength of carbapenemase production by the test strains. In addition, you only need to input the OD values to the model and the result is the number of carbapenemase producers. The data can be output directly to reduce the time for manual entry and analysis of data. Lastly, the model gives a standard procedure for reading experimental results of carbapenemase producers and climates reading errors. In the next step, we will create a website platform that co-networking with hospitals and research laboratories, the results of carbapenemase producers in different areas can be compared to effectively monitor the prevalence of carbapenemases in the region. By providing actionable data directly to governments, a website that could analyze those areas containing CPGNB could forewarn healthcare facilities to take the appropriate infection control measures.

We evaluated a deep learning method (AI-Blue-Carba) which allows detection of CPGNB in less than 15 min. This test can be used as the first step in detecting the carbapenemase activity of candidate isolates. In addition, it is also useful for checking carbapenemase activity as part of the infection control process for outbreaks caused by carbapenemase producers. AI-Blue-Carba is a robust assay which user-friendly (no need for trained staff), high in performance (sensitive and specific), and low in cost.

## Data Availability Statement

The original contributions presented in the study are included in the article/Supplementary Material, further inquiries can be directed to the corresponding authors.

## Author Contributions

JS and X-ML conceived of the study. LJ, LH, and S-CB performed the experiments. LJ and H-XC constructed models. LJ, Z-HC, R-SY, and R-MZ analyzed the data. LJ, X-WL, and RW made the figures. LJ wrote the manuscript. JS, LC, X-ML, and X-PL edited and revised the manuscript. Y-HL coordinated the whole project. All authors have read and approved the final manuscript.

## Conflict of Interest

The authors declare that the research was conducted in the absence of any commercial or financial relationships that could be construed as a potential conflict of interest.
