# Supplementary material for: AI-Blue-Carba: A Rapid and Improved Carbapenemase Producer Detection Assay Using Blue-Carba With Deep Learning
Source: Front Microbiol. 2020 Nov 20;11:585417. doi: 10.3389/fmicb.2020.585417 (PMC7714720; doi:10.3389/fmicb.2020.585417)
Supplement: Supplementary file 1 [file Table_1.docx]

**Table S1.** The sensibility and specificity of AI-Blue-Carba and Blue-Carba from different time group

|  | AI-Blue-Carba | |  | Blue-Carba | |
| --- | --- | --- | --- | --- | --- |
| Time Group | sensibility | specificity |  | sensibility | specificity |
| 0-5min | 94.4% | 82.6% |  | 82.2% | 100% |
| 0-10min | 95.3% | 95.7% |  | 90.6% | 100% |
| 0-15min | 95.3% | 95.7% |  | 92.5% | 100% |
| 0-20min | 96.3% | 95.7% |  | 92.5% | 100% |
| 0-25min | 96.3% | 100% |  | 92.5% | 100% |
| 0-30min | 96.3% | 100% |  | 93.5% | 100% |
| 0-35min | 96.3% | 100% |  | 96.5% | 100% |
| 0-40min | 96.3% | 100% |  | 96.5% | 100% |
| 0-45min | 96.3% | 100% |  | 96.5% | 100% |
| 0-50min | 96.3% | 100% |  | 96.5% | 100% |
| 0-55min | 96.3% | 100% |  | 96.5% | 100% |
| 0-60min | 96.3% | 100% |  | 96.5% | 100% |

**Figure S1**. Determined the optimum wavelength of yellow and blue. A. Scanned the optimum wavelength of positive result of Blue-Carba(yellow); B. Scanned the optimum wavelength of negative result of Blue-Carba(blue).

**Figure S2.** Determined the diluent and bacterial concentration of AI-Blue-Carba. A-C and D-F were diluted by PBS and ddH_2_O, respectively. The bacterial concentration of A and D, Band E, and C and F were 1.0 OD, 1.5 OD, 2.0 OD, respectively.
