# Supplementary figures and images for: AI-Blue-Carba: A Rapid and Improved Carbapenemase Producer Detection Assay Using Blue-Carba With Deep Learning
Source: Front Microbiol. 2020 Nov 20;11:585417. doi: 10.3389/fmicb.2020.585417 (PMC7714720; doi:10.3389/fmicb.2020.585417)

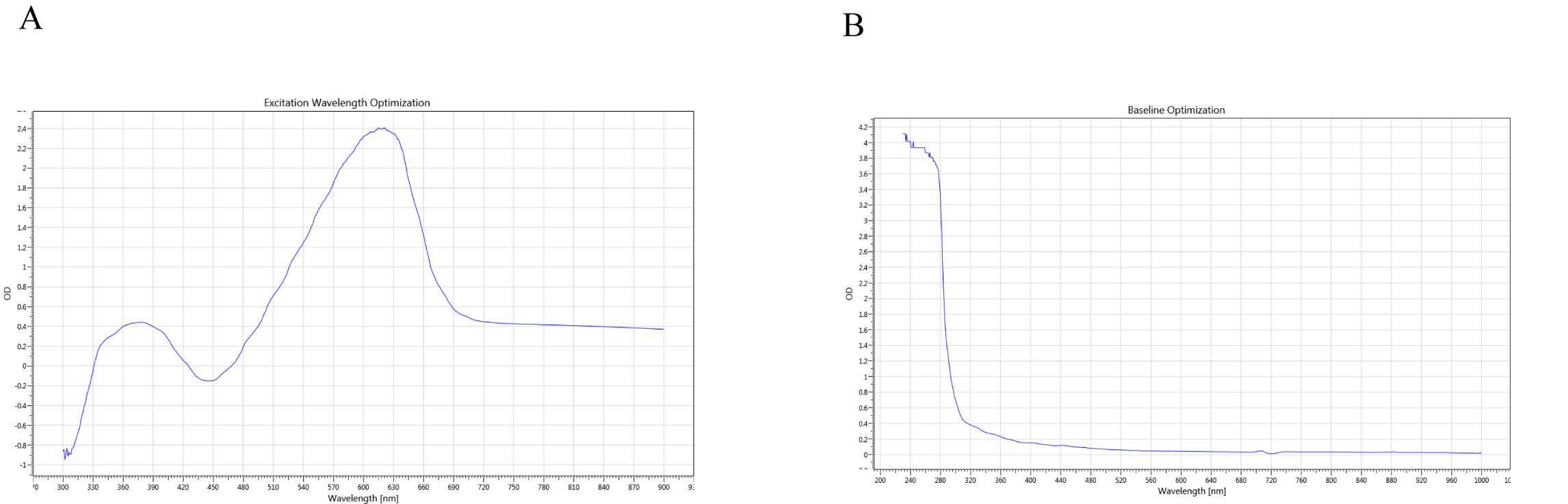

Supplement: Supplementary file 2 [file Image_1.TIF]

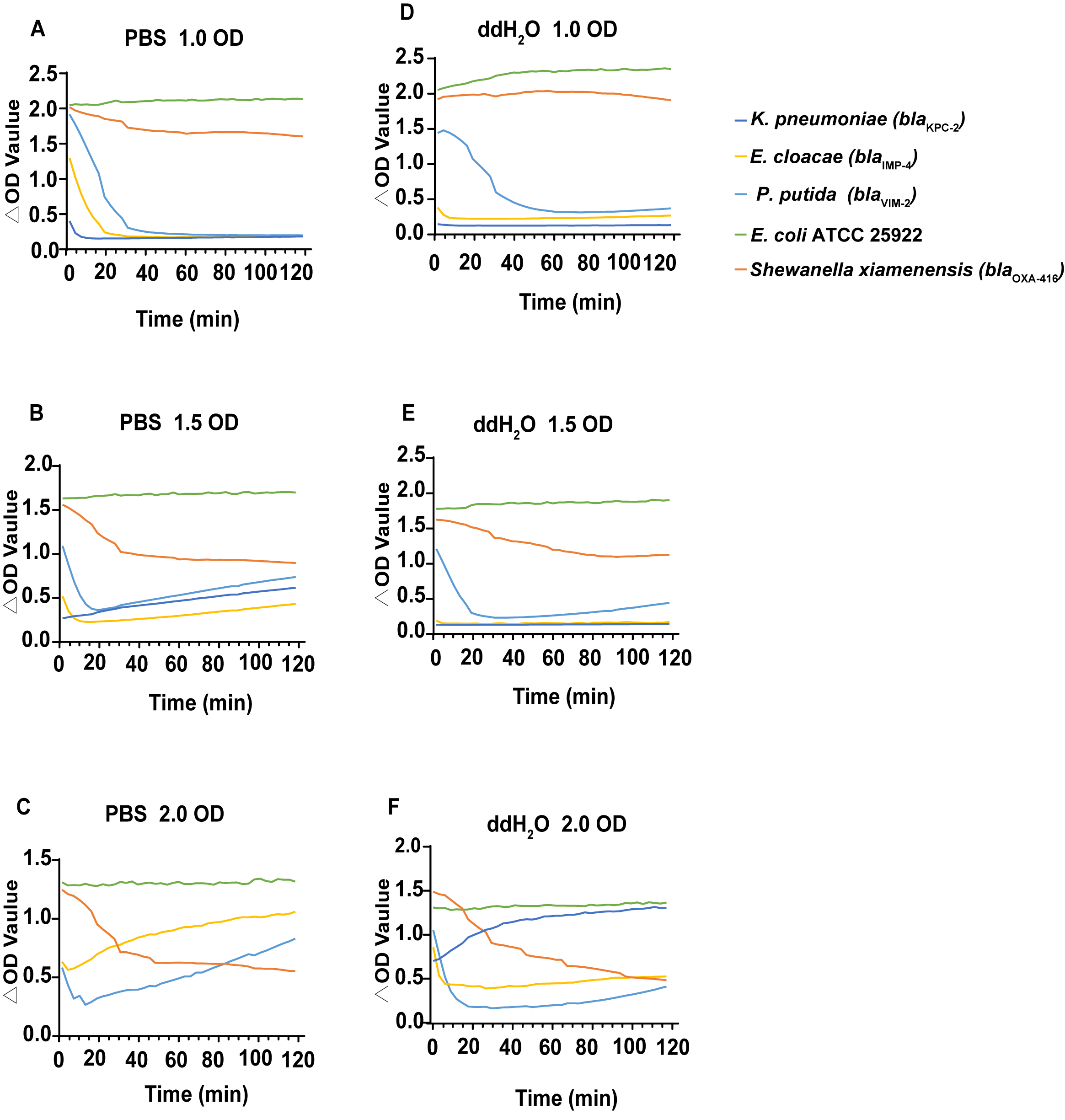

Supplement: Supplementary file 3 [file Image_2.TIF]
